# Measuring neighborhood climate and public stigma among veterans with mental illnesses

**DOI:** 10.1186/s12888-026-08075-0

**Published:** 2026-05-28

**Authors:** Emre Umucu, Guillermina R. Solis, Rebecca Campa, Aylin Celik Zencir, Andrew Vernon, Muharrem Koc, Beatrice Lee

**Affiliations:** 1 Department of Public Health Sciences, College of Health Sciences, The University of Texas at El Paso, El Paso, TX USA; 2 Director of CHS REACHED, College of Health Sciences, The University of Texas at El Paso, El Paso, TX USA; 3 Audie L. Murphy Memorial VA Hospital, South Texas VA Medical Center, San Antonio, TX USA; 4 College of Nursing, The University of Texas at El Paso, El Paso, TX USA; 5 Department of Rehabilitation Psychology and Special Education, The University of Wisconsin Madison, Madison, WI USA; 6 Interdisciplinary Health Sciences Doctoral Program, College of Health Sciences, The University of Texas at El Paso, El Paso, TX USA; 7 Rehabilitation Sciences Program, College of Health Sciences, The University of Texas at El Paso, El Paso, TX USA

**Keywords:** Veterans, Mental illness, Public stigma, Neighborhood climate, Self-determination theory, Autonomy support

## Abstract

**Background:**

This study, grounded in Self-Determination Theory (SDT), explores the role of autonomy-supportive neighborhoods in reducing public stigma among veterans with mental illnesses. It aims to (1) validate the Neighborhood Climate Questionnaire (NCQ) and (2) evaluate the relationship between neighborhood climate, mental health symptoms, and public stigma within a sample of U.S. veterans with mental illness.

**Methods:**

A cross‑sectional sample of 156 U.S. veterans with self‑reported mental illnesses completed measures of neighborhood climate, well‑being, depression, functional limitations, and public stigma. An exploratory factor analysis and Cronbach’s alpha were used to examine the psychometric properties of NCQ. Hierarchical multiple regression analysis examined the association between neighborhood climate and public stigma after controlling for demographic and clinical variables.

**Results:**

Exploratory factor analysis supported a one‑factor structure of the NCQ, accounting for 84% of the variance, with excellent internal consistency (α = 0.97). In regression analysis, greater autonomy‑supportive neighborhood climate was significantly associated with lower public stigma (β = −0.21, *p* < .05), beyond demographic characteristics, functional limitations, and depression.

**Conclusion:**

These findings highlight the need for community-based interventions to foster autonomy support, improve social integration, and reduce stigma-related barriers to mental health care. Future research should explore regional differences and longitudinal effects.

**Trial registration:**

N/A.

**Clinical trial number:**

NA.

## Introduction

Mental illness is an ongoing global public health concern. According to World Health Organization (WHO), more than one billion individuals living with mental health conditions (World Health Organization [32]). Veterans, in particular, experience disproportionately higher rates of mental illness compared to civilians, with conditions such as post-traumatic stress disorder (PTSD), depression, and suicidal ideation being more prevalent [10, 24, 25]. Contributing factors include combat exposure, stigma associated with seeking help, reintegration challenges, and adverse childhood experiences [1].

Public stigma, the negative public attitude leading people to distance themselves from those with mental illness, exacerbates mental health challenges by discouraging help-seeking behavior and increasing distress [9, 17, 26]. Among veterans, stigma is often linked to feelings of weakness and embarrassment, further discouraging engagement with mental health services [17]. Research indicates that stigma leads veterans to avoid mental health treatment, leading to untreated conditions and worsening symptoms [21].

Despite these challenges, certain protective factors such as autonomy-supportive environments can buffer against the negative effects of stigma. Autonomy-supportive environments are settings in which people feel that their choices are respected, and they have meaningful input into matters that affect their daily lives, thus fostering a sense of belonging. In the neighborhood context, autonomy-supportive neighborhoods may be reflected in features such as inclusive community decision-making, nonjudgmental community discussions, neighbors who encourage help-seeking rather than discriminate it, and social norms that value different voices in backgrounds and mental health experiences. Among civilians, social support from peers, friends, and family members plays a critical role in mitigating the impact of mental illness and reducing stigma-related barriers to care [2, 15]. Social connections within a veteran’s neighborhood can enhance well-being and reduce psychological distress [8]. The concept of basic psychological need support, one of the key tenets of Self-Determination Theory (SDT), is particularly relevant in this context [22].

SDT posits that autonomy, competence, and relatedness are fundamental psychological needs that contribute to motivation and well-being [4, 22]. Autonomy refers to the experience of acting with a sense of volition and in accordance with one’s values. Autonomy-supportive environments may foster these needs through encouragement, choice, and respect [31]. Competence refers to a sense of effectiveness, and competence support often entails having access to information and resources [4]. Finally, relatedness refers to a feeling connection and belonging and is fostered by inclusion [4]. Studies have shown that psychological support from healthcare providers and other significant individuals can enhance perceived competence and foster internalization of health-related goals [31].

Growing evidence suggests that neighborhood environments may play a critical role in shaping these experiences, especially public stigmas. Research revealed that the quality of relationships between neighbors have impact on individuals’ mental health [14]. For example, ongoing exposure to neighborhood challenges can create chronic stress and isolation [28]; while supportive relationships with neighbors can foster better mental health by offering emotional support [12, 23]. However, empirical tools to assess neighborhood climate in people with mental illness and Veteran populations remain limited. This study aims to fill this gap by examining the relationship between neighborhood climate and public stigma among veterans with mental illness.

The *Neighborhood Climate Questionnaire* (NCQ) was adapted and validated to measure perceptions of autonomy support among persons with mental illness and Veterans. Validating the NCQ for these populations is essential since measuring neighborhood climate accurately can help us capture neighborhood-level factors that influence mental health engagement and outcomes. Specifically, we aim to (1) validate NCQ for use in this population and (2) assess the impact of autonomy-supportive neighborhood climates on veterans’ perceptions of public stigma. We hypothesized that the NCQ would demonstrate a unidimensional factor, that higher perceived neighborhood autonomy support would be positively associated with well-being and negatively associated with depressive symptoms, and that, after accounting for demographic and clinical characteristics, more autonomy-supportive neighborhood climates would predict lower levels of public stigma among veterans.

Findings from this study may inform the development of community-based interventions designed to foster autonomy support, enhance well-being, and reduce stigma-related barriers to care. Based on these objectives, we hypothesized that (a) the NCQ would demonstrate a unidimensional factor structure, reflecting a single underlying construct of perceived neighborhood autonomy support; (b) higher NCQ scores would be positively associated with well-being and negatively associated with depression; and (c) after controlling for demographic variables (age, gender, race) and clinical factors (functional limitations, depression), neighborhood climate would significantly predict lower levels of public stigma.

## Methods

### Procedure

The study was conducted in accordance with the Declaration of Helsinki and approved by the Institutional Review Board of the University of Texas at El Paso (ID#1153068-1). All participants completed an online consent to voluntarily participate in the study. Participants were recruited through Amazon’s Mechanical Turk (MTurk), a widely recognized and reliable platform for data collection [20]. As noted by Goodman et al., [7], MTurk offers scholars access to a broad and diverse participant pool while also allowing for the implementation of selection criteria, such as geographic restrictions. Our study participation was restricted to U.S. residents who had previous military service. This study included only veterans who (a) were 18 years or older and (b) self-reported having a mental illness.

### Participants

The study included a total of 156 veterans diagnosed with mental illnesses (*M*_age_ = 37.99, SD = 10.64). The sample was predominantly male (66.7%) and White (70%), with over half of the participants (53.3%) being married. Regarding education, 50.7% had at least a bachelor’s degree. In terms of military branch, the majority served in the Army (56%), followed by the Air Force (18%), Navy (12.7%), Marine Corps (10%), and Coast Guard (3.3%). Additionally, 62% of participants had served in Operation Enduring Freedom and Operation Iraqi Freedom. The average number of combat tours was 1.90 (SD = 2.31). A total of 64 participants (42.7%) reported having a service-connected disability rating. All participants had at least one diagnosed mental illness, with the most common being depression (64.7%), followed by anxiety (58.7%), PTSD (34.7%), substance use disorder (10.7%), bipolar disorder (4.7%), personality disorder (4.7%), and schizophrenia (1.3%). Please see Table 1 for the details.

### Materials

#### Neighborhood climate

The *Neighborhood Climate Questionnaire* (NCQ) was developed by adapting items from the *Health Care Climate Questionnaire* [30, 31]. The original HCCQ measures perceived autonomy support provided by health-care practitioners. To align with the study’s focus on neighborhood climate and community participation, the wording of each item was systematically modified to reflect interactions with neighbors rather than health-care providers, and to emphasize community activities instead of health-related behaviors. Each item is rated on a 7-point Likert-type scale ranging from one (strongly disagree) to seven (strongly agree), with higher scores representing higher levels of autonomy support received from neighbors. The original HCCQ has been applied in multiple studies, demonstrating reliability coefficients (Cronbach’s alpha) between 0.92 and 0.96 [30, 31].

#### Well-being

Participants’ well-being was measured using the *PERMA-Profiler* [3]. The PERMA-Profiler measures five dimensions (i.e., positive emotion, engagement, relationships, meaning, accomplishment) of the well-being proposed by the Seligman (2011). Each dimension has a total of three items totaling 15 items rated on an 11-point scale ranging from 0 (never) to 10 (always) or 0 (not at all) to 10 (completely). The reported Cronbach’s alpha scores ranged from 0.71 to 0.89 for positive emotion, 0.60 to 0.81 for engagement, 0.75 to 0.85 for relationships, 0.85 to 0.92 for meaning, and 0.70 to 0.86 for accomplishment subscale scores [3]. In the current study, the PERMA-Profiler was found to have a 0.96 internal consistency reliability coefficient.

#### Public stigma

Participants’ public stigma was measured using adaptation [5] of the *Discrimination Devaluation Scale* [16]. The scale consists of a total of 12 items, rated on a 6-point Likert scale ranging from zero (strongly disagree) to five (strongly agree), with higher scores representing higher levels of public stigma. The revised scale showed strong internal consistency, with a Cronbach’s alpha of 0.89, reflecting reliable responses across items [5]. In this study, the internal consistency reliability coefficient was 0.89.

#### Functional disability

Participants’ functional limitations were measured using *the World Health Organization Disability Assessment Schedule II* (WHODAS 2.0) [27]. This 12-item scale rates disability levels on a 1 (none) to 5 (extreme or unable to perform) scale. The authors reported exceptional internal consistency for the mental health disability subgroup, with an overall Cronbach’s alpha of 0.98 [27]. We used the total score, where higher scores indicate greater functional limitations. In this study, the internal consistency reliability coefficient was 0.91.

#### Depression

Depression was assessed utilizing the *Patient Health Questionnaire 9* (PHQ-9) [13]. The scale comprises nine items, rated on a 0 (not at all) to 3 (nearly every day) scale. The Cronbach’s α of the PHQ-9 measure was reported to range between 0.86 and 0.89 [13]. The internal consistency reliability coefficient for the PHQ-9 was 0.90.

### Data analysis

For the first purpose of this study, an exploratory factor analysis (EFA) was first conducted to understand the factor structure of the NCQ. Prior to analysis, data were screened for missingness; cases with more than 10% missing responses were excluded. Assumptions of factor analysis were evaluated, including sampling adequacy and sphericity, including the Kaiser–Meyer–Olkin (KMO) measure and Bartlett’s test of sphericity. The internal consistency reliability of the NCQ was assessed using Cronbach’s alpha (α). To establish concurrent validity, Pearson correlation coefficients were computed between NCQ scores and measures of well-being and depression. For the second purpose of this study, a hierarchical multiple regression analysis was performed to examine the relationship between neighborhood climate and public stigma. Age, gender, and race were entered as control variables in Step 1; functional limitations and depression were entered in Step 2; and neighborhood climate was entered in Step 3. Analyses were conducted using SPSS 31, and statistical significance was set at *p* < .05.

## Results

### EFA

An exploratory factor analysis (EFA) was conducted to examine the factor structure of the Neighborhood Climate Questionnaire (NCQ). Principal axis factoring was used as the extraction method. Preliminary analyses indicated that the data were suitable for factor analysis. The Kaiser–Meyer–Olkin (KMO) measure of sampling adequacy was 0.91, exceeding recommended criteria, and Bartlett’s test of sphericity was statistically significant, χ²(15, *N* = 156) = 1187.45, *p* < .001, indicating that the correlation matrix was appropriate for factor analysis. Both the Kaiser–Guttman criterion (eigenvalues > 1.0) and inspection of the scree plot supported a single-factor solution. The extracted factor accounted for 83.52% of the total variance. All six items loaded strongly on the single factor, with standardized factor loadings ranging from 0.87 to 0.94 (Table 1). Because only one factor was extracted, rotation was not applicable.

### Reliability

The internal consistency reliability coefficient (Cronbach’s α) for the NCQ was computed to be 0.97, indicating excellent reliability in a sample of veterans with mental illness.

### Concurrent validity

The NCQ was positively associated with well-being scores (*r* = .42, *p* < .001). Conversely, the NCQ was negatively associated with depression (*r* = − .18, *p* < .001). These findings provide evidence for the concurrent validity of the NCQ.

### Association between neighborhood climate and public stigma

A hierarchical multiple regression analysis was conducted to examine whether perceived neighborhood climate was associated with public stigma after controlling for demographic and clinical variables (Table 2). In Step 1, age, gender, and race were entered into the model and did not account for a significant proportion of variance in public stigma (R² = 0.006, F(3, 152) = 0.32, *p* = .81). In Step 2, functional limitations and depressive symptoms were added, resulting in a significant increase in explained variance (ΔR² = 0.057, F-change(2, 150) = 4.58, *p* = .012). Within this model, depressive symptoms were a significant positive predictor of public stigma (β = 0.28, *p* = .005), whereas functional limitations were not significant. In Step 3, neighborhood climate was entered into the model and explained a significant additional proportion of variance in public stigma (ΔR² = 0.042, F-change(1, 149) = 7.01, *p* = .009). Greater autonomy-supportive neighborhood climate was significantly associated with lower levels of public stigma (β = −0.21, *p* = .009). The final model was statistically significant (F(6, 149) = 2.93, *p* = .010) and explained 10.5% of the variance in public stigma (R² = 0.105).

## Discussion

The first purpose of this study was to validate the NCQ, which is adapted from the *Healthcare Climate Questionnaire* [30], for the veterans with mental illness. Results supported the NCQ as a reliable and valid measure, demonstrating a unidimensional factor structure, excellent internal consistency (α = 0.97), and significant concurrent validity, with positive correlations with well-being (*r* = .42) and negative correlations with depression (*r* = − .18). The second and most important objective was to examine the relationship between neighborhood climate and public stigma. Our findings revealed a significant negative association between need-supportive neighborhood climate and perceived public stigma. These results align with previous research by Weinstein et al., [29], which emphasized the critical role of need supports in enhancing well-being among stigmatized individuals.

Veterans with higher levels of perceived neighborhood support reported lower levels of public stigma. This underscores the value of community environments that foster choice, respect, and inclusion. Previous literature shows that public stigma can discourage veterans from seeking help [17, 18, 21] and negatively impact physical and mental health [33]. Our findings suggest that basic psychological need-supportive neighborhood climates may counter these negative outcomes by enhancing motivation and facilitating engagement with mental health resources [6, 11]; however, this association should be further explored in future intervention-based research.

Furthermore, lower levels of support were associated with higher levels of perceived public stigma, which contributes to social isolation and negatively impacts well-being [19]. These results indicate that neighborhoods fostering greater autonomy competence and relatedness may also promote inclusivity and decrease stigmatizing attitudes toward mental illness. Such environments may play a critical role in promoting social connection and psychological resilience among veterans with mental illnesses.

Our findings have several practical implications. First, clinicians could utilize the NCQ to assess perceptions of neighborhood climate and predict stigma-related barriers faced by veterans with mental illness. This can guide targeted interventions that foster empowerment and address stigma associated with lower levels of positive neighborhood climate. Second, our results support the development of community-based programs that create need-supportive environments, such as veteran peer support programs or neighborhood networks. For example, veterans enrolled in VA benefits may receive peer support services, which can improve their health and well-being outcomes. Besides, peer support services can help Veterans with mental illness see that recovery is possible. Assigning peer support specialists to lead these initiatives could enhance their effectiveness by providing relatable, autonomy-supportive guidance. This may be particularly beneficial in rural settings, where access to formal mental health resources is limited, and informal community support structures may fill the gap. It is also important to interpret these findings in light of broader community context. Objective neighborhood characteristics, such as socioeconomic disadvantage, may shape both perceived neighborhood climate and stigma, creating potential unmeasured confounding. Accordingly, future research should include perceived neighborhood climate with objective neighborhood indicators to disentangle individual-level perceptions from contextual neighborhood conditions.

Despite the strengths of the current study, several limitations must be recognized. Although the NCQ demonstrated excellent psychometric properties, we did not conduct a confirmatory factor analysis, which should be addressed in future research. Additionally, the study design was cross-sectional, limiting our ability to draw causal conclusions. Data was self-reported and collected online, which could introduce response bias and exclude individuals without reliable internet access. A key limitation is that we did not measure general perceived social support (e.g., support from family, friends, or peers) using a dedicated instrument. As a result, we cannot rule out the possibility that broader social support partially explains the observed association between neighborhood climate and public stigma. Our study did not include objective neighborhood-level indicators such as income, crime rates, or other community-level socioeconomic data, which may confound perceptions of neighborhood climate. Future research should investigate how regional differences shape the relationship between neighborhood climate and public stigma. Longitudinal designs would be valuable in establishing causality and examining the impact of neighborhood climate over time. Further validation of the NCQ through confirmatory factor analysis and testing across diverse populations would also strengthen its utility.

Overall, this study contributes to the literature by providing psychometric validation of the *Neighborhood Climate Questionnaire* among veterans with mental illnesses and by demonstrating the importance of autonomy-supportive neighborhood environments in shaping perceptions of negative attitudes. By extending SDT to the neighborhood context, the findings highlight community-level psychological need support as a potentially malleable factor influencing negative attitudes and social inclusion. Future research should employ longitudinal and intervention-based designs to examine causal mechanisms and explore how neighborhood-level interventions may foster autonomy support and reduce stigma across diverse veteran populations.

**Table 1:** Factor matrix, communalities, means, and standard deviations of items and total score, with reliability information

Item	Factor loading	h^2^	M	SD	α if item deleted
My neighbors encourage me to ask questions about community activities in terms of improving my community participation.	0.944	0.891	3.45	2.02	0.959
My neighbors try to understand how I see community activities in terms of improving my community participation before suggesting any.	0.931	0.868	3.40	2.03	0.960
My neighbors listen to how I would like to do things regarding community activities in terms of improving my community participation.	0.926	0.857	3.38	1.89	0.961
My neighbors convey confidence in my ability to make changes regarding community activities in terms of improving my community participation.	0.910	0.828	3.52	1.94	0.962
I feel my neighbors have provided me with choices and options about community activities in terms of improving my community participation.	0.896	0.804	3.42	1.94	0.963
I feel my neighbors understand how I see things with respect to community activities in terms of improving my community participation.	0.874	0.764	3.38	1.89	0.966
**Total ** ***M*** ** and ** ***SD***	3.24 (1.81)				
**Eigenvalue**	5.01				
**% variance**	83.52				
**Cronbach’s alpha**	0.97				

**Table 2:** Hierarchical multiple regression analysis predicting public stigma

Variable	β	*R* ^2^	ΔR^2^	F	F_change_
**Step 1**		0.006	0.006	0.32	0.32
Age	−0.04^†^				
Gender^a^	−0.02^†^				
Race^a^	0.06^†^				
**Step 2**		0.063	0.057	2.03	4.58^*^
Functional Limitations	− 0.09^†^				
Depression	0.28^*^				
**Step 3**		0.105	0.042	2.93	7.01^*^
**Neighborhood Climate**	− 0.21^*^				

**a**Gender (male = 1), race (White = 1). †*p* = *ns*. *p < .05

## Data Availability

The datasets generated and/or analysed during the current study are not publicly available due ethical reasons but are available from the corresponding author on reasonable request.
